# Supplementary material for: Physical Exercise and the Gut Microbiome: A Bidirectional Relationship Influencing Health and Performance
Source: Nutrients. 2024 Oct 28;16(21):3663. doi: 10.3390/nu16213663 (PMC11547208; doi:10.3390/nu16213663)
Supplement: Supplementary file 1 [file nutrients-16-03663-s001.zip › nutrients-3238063-supplementary.pdf]

Table S1: Studies investigating effects on gut microbiome following intervention with exercise in animals.

**Study design and country**	**Animal characteristics<br>Sample size and sex (n, M/F)<br>Age (mean ± SD or median [IQR], years)<br>BMI (mean ± SD or median [IQR], kg/m²)**		**Intervention**	**Duration**	**Effects**	**References**
	**Control**	**Intervention**				
Interventional<br>US	F344 rats (40, M)	F344 rats (40, M)	Voluntary wheel running	6 weeks	↑*Bacteroidetes*, other Bacterial genera<br>↓*Firmicutes*	[1]
Interventional<br>Japan	Winstar rats (7, M)	Winstar rats (7, M)	Voluntary wheel running	5 weeks	↑n-butyrate, *Firmicutes*	[2]
Interventional<br>Poland	Thoroughbred racehorses (17)<br>Mares (7), Stallions (10)	Thoroughbred racehorses (17)<br>Mares (7), Stallions (10)	1900m race	6 weeks	↑*Bacteroidetes, Firmicutes*	[3]
Interventional<br>USA	C57BL/6 mice	C57BL/6 mice	Low fat diet, high fat diet, sedentary, exercise (voluntary wheel running)	5 weeks	↑Soleus:body weight, Heart: body weight, diversity of bacteria in high fat exercise intervention mice, *Bacteroidetes*<br>↓*Firmicutes, Actinobacteria*	[4]
Interventional<br>USA	C57BL/6 mice<br>11-13 months age	C57BL/6 mice<br>11-13 months age	Voluntary wheel running	5 weeks	↑ *Firmicutes-Bacilli-Lactobacillales*<br>↓*Tenericutes, Bacteroidetes*	[5]
Interventional<br>USA	6-week-old C57BL/6NTac mice (36, M)	6-week-old C57BL/6NTac mice (36, M)	Voluntary wheel running	12 weeks	↓inflammatory infiltrate<br>↑GM diversity, *Allobaculum, Clostridiales*<br>*F. prausnitzii* only detected in exercised animals	[6]
Interventional<br>Brazil	45-day old Wistar rats (5, M)<br>216.5 +- 10.9 grams	45-day old Wistar rats (5, M)<br>216.5 +- 10.9 grams	Resistance training	12 weeks	↑ α diversity, *Firmicutes, Bacteroidetes*	[7]

					β diversity indicated a distinct bacterial community in the resistance training group	
Interventional<br>China	Female mice<br>3-4 weeks old<br>C57BL/6 WT mice	Female mice<br>3-4 weeks old<br>C57BL/6 WT mice	Strength exercise	4 weeks	↑*Bacteroidetes, Verrucomicrobia-Akkermansia, Clostridium, Parabacteroides, Christensenella, Dorea, Roseburia, Paraprevotella*<br>↓*Firmicutes, Anaerotruncus, Jeotgalicoccus, Anaerotruncus, Alistipes, Ruminococcus, Desulfovibrio*	[8]
Interventional<br>Italy	Male rats<br>3 months old<br>Wistar rats<br>300g weight	Male rats<br>3 months old<br>Wistar rats<br>300g weight	Chow-fed or food withdrawal<br>Exercise or no exercise	3 days	↓α diversity in food-withdrawn and food-withdrawn/exercise groups	[9]
Interventional<br>China	4 weeks old before 12-week feeding intervention, 16-weeks old when given exercise and cold intervention<br>40 Sprague-Dawley rats	4 weeks old before 12-week feeding intervention, 16-weeks old when given exercise and cold intervention<br>40 Sprague-Dawley rats	High-fat high-sugar diet for 12 weeks<br>Exercise or no exercise<br>Varying levels of cold exposure	12 weeks	↑*Verrucomicrobia, Deferribacteres, , Firmicutes, Saccharibacteria,, Cyanobacteria* from cold exposure<br>Exercise reversed the increase in all these phyla<br>↑*Proteobacteria*	[10]
Interventional<br>USA	Rats<br>Young rats and adult rats	Rats<br>Young rats and adult rats	Voluntary wheel running	8 weeks	No change in the diversity of the gut microbiome	[11]
Interventional<br>Brazil	Male mice<br>4 weeks old	Male mice<br>4 weeks old	Normal diet and high-fat diet that induced	4 months of diet	↓*Proteus*<br>↑*Vagococcus*	[12]

	C57BL6 mice	C57BL6 mice	obesity<br>Exercise or no exercise	4 weeks of training		
Interventional<br>Brazil	Mangalarga Marchador fillies (12)<br>2.5 to 3 years old<br>330 +- 30 kg weight	Mangalarga Marchador fillies (12)<br>2.5 to 3 years old<br>330 +- 30 kg weight	Exercise or no exercise<br>Supplementation with carnitine or chromium	5 weeks	↓fecal pH, *Proteus*<br>↑SCFAs, *Vagococcus*	[13]
Interventional<br>UK	Male PolgA mutant mice<br>4 months old	Male PolgA mutant mice<br>4 months old	Sedentary or exercise	7 months	↑*Mucispirillum, Desulfovibrio, Bacteroides,* temporally<br>↓*Helicobacter, Lactobacillus,* temporally	[14]
Interventional<br>Ireland	Mixed breeds (Cob, Pony, Cob Pony, SB) (14, M & F)<br>4-17 years old<br>500 kg weight	Mixed breeds (Cob, Pony, Cob Pony, SB) (14, M & F)<br>4-17 years old<br>500 kg weight	Control group: 2% bodyweight diet and regular walking<br>Treatment group: 1.4% bodyweight diet and exercise program<br>Treatment group split into two groups, accounting for length of stride (>148 cm stride)	6 weeks	↑α, β diversity, *Coprococcus, Clostridia*<br>↓*Eubacteriaceae, Pseudomonadaceae*	[15]
Interventional<br>Canada	C57BL mice (M)<br>6 weeks old	C57BL mice (M)<br>6 weeks old	Type 2 diabetic or normal mice<br>Sedentary or Exercise	6 weeks	↑ *Lactobacillus, Clostridium leptum*<br>↓Total bacteria, *Bacteroides/Prevotella, Methanobrevibacter*<br>↑*Bifidobacteria* in nondiabetic mice	[16]

Table S2: Studies investigating effects on gut microbiome based on General Physical activity (PA) and Cardiorespiratory Fitness (CRF).

**Study design and country**	**Participant characteristics<br>Sample size and sex (n, M/F)<br>Age (mean ± SD or median [IQR], years)**	**Intervention**	**Duration**	**Effects**	**References**

	BMI (mean ± SD or median [IQR], kg/m^2)					
	**Control**	**Intervention**				
Observational Spain	Sedentary women (21, F) 32.2±8.7 22.9±3	Active women (19, F) 30.7±5.9 24.4±4.5	PA	1 week	↑*Firmicutes-Faecalibacterium prausnitzii, Roseburia hominis, Verrucomicrobia-Akkermansia muciniphila), Actinobacteria-Bifidobacterium, Proteobacteria-Haemophilus, Bacteroidetes-Paraprevotella*	[17]
Observational US		Community dwelling men (373, M) 84±3.9 26.9±3.8	PA		↑β diversity, *Cetobacterium* ↓*Coprobacillus, Adlercreutzia, Erysipelotrichaceae*	[18]
Observational Spain	Sedentary (45) 33.69±7.96 23.63±2.91	Active (64) 32.17±7.4 24.01±3.28	PA		↑*Roseburia faecis*, *Roseburia* ↓*Sutterella*	[19]
Observational US		Breast cancer survivors (12, F) 55±13 30.4±5.1	30′ vigorous/60′ moderate PA Eventually increase to ≥150’ moderate	3 months	↑β diversity with CRF, *Roseburia*, *Clostridiaceae*	[20]
Observational Italy		University students (140, 68M/72F) 22.5±2.9 22.4±2.8	PA		↑β diversity ↓*Firmicutes (Megasphaeram Lachnobacterium, Dialister), Bacteroidetes (Paraprevotella*)	[21]
Observational US		Healthy individuals (3409, 1398M/2011F) 49±12 27±6	PA		↑α index ↑*Firmicutes (Ruminococcaceae, Clostridiales, Veillonella, Lachnospira, Faecalibacterium)*	[22]
Observational US		College students (82, 35M/47F) 18.4±0.6 24.4±5.5	PA		↑*Paraprevotellaceae, Lachnospiraceae, Lachnospira* ↓*Enterobacteriaceae, Enterobacteriales*	[23]
Observational Denmark		Community dwelling subjects (207,	PA		↑α, β diversity ↑*Bifidobacteriales, Clostridiales*	[24]

		109M/98F)<br>70.2±3.9<br>25.7±3.8				
Observational<br>China		Adults (1589), Elderly (897)<br>18-60, 60+<br>413 overweight, 22 underweight-Elderly	Daily, regular, occasional, rare PA		↑*Actinobacteria-Eggerthellaceae, Bifidobacteriacease, Clostridiaceae*:<br>Overweight: ↑α diversity, *Bacteroidetes, Cyanobacteria, Firmicutes, Tenericutes, Verrucomicrobia, Turicibacteraceae*<br>↓*Pseudomonadaceae, Proteobacteria-Oxalobacteraceae, Odoribacteraceae, Barnesiellaceae*	[25]
Observational<br>US		Healthy children (267, 178M/89F)<br>11.5±3.8<br>43 overweight, 56 underweight	PA		↑α diversity	[26]
Observational<br>China		Healthy participants (131, 80M/51F)<br>35.8±12<br>16-35	PA	1 month	↓α diversity	[27]
Observational<br>US		Breast cancer survivors (37, F)<br>55±9<br>31.8±7.8	PA	10 days	↑α, β diversity with CRF	[28]
Observational<br>Taiwan		Normal weight group (32, 17M/15F)<br>68.9±9.1<br>21.8±2<br>Normal weight obese group (32, 17M/15F)<br>67.5±10.9<br>22.1±1.6<br>Overweight group (32, 17M/15F)<br>67.8±10.7<br>27.8±2.5	PA	1 week	↑α diversity, *Coprococcus*	[29]
Observational	Normal weight	Overweight (46,	PA	1 week	↓*Firmicutes-Megamonas, M. funiformis, Megasphaera, M. hominis,*	[30]

Italy	(46, 5M/41F)<br>49±11<br>21.6±2.1	6M/40F)<br>50±12<br>36.6±6			*Eubacterium biforme*	
Observational<br>Netherlands		Active participants (868, 426M/442F)<br>53.4±10.5<br>26.7±4.5	At least 150 min of PA per week		↑α diversity, *Lachnospiraceae, Veillonella*	[31]
Observational<br>US		FHS Generation 3 (1423, 630M/793F)<br>55<br>28	PA	1 month	↑α diversity	[32]
Observational<br>US	Without PA ≥ 60 min (132, 66M/66F))<br>10<br>51 overweight	PA ≥ 60 min (189, 108M/81F)<br>10<br>57 overweight	PA	1 year	Moderate PA associated with ↑α diversity in Females<br>↑*Christensenellaceae*, *Subdoligranulum*<br>↓*Blautia, Faecalibacterium, Roseburia, Ruminococcaceae*	[33]
Observational<br>Israel	Less active with insomnia (18, 4M/14F)<br>73.66±6.65<br>27.756 ± 3.27	More active with insomnia (31, 6M/25F)<br>72.22±5.08<br>25.81 ± 3.94	PA<br>More active=daily step counts≥6500	2 weeks	↑*Bifidobacterium, Clostridium, Catenibacterium, Peptococcus, Holdemanella, Butyricicoccus*<br>↓*Barnesiella, Blautia, Lachnoclostridium,* Christensenellaceae	[34]
Observational<br>Taiwan		Healthy participants (119, 38M/81F)<br>47.1±15<br>38 overweight	PA		high to moderate PA: ↑*Faecalibacterium-F. prausnitzii*	[35]
Observational<br>US		Prostate cancer patients (40, M)<br>60±7<br>30.1±3.1	PA		↑*Firmicutes*	[36]
RCT<br>Denmark		High intensity participants (24, 12M/12F)<br>39 [33-42]<br>29.9 [28.2-32.1]	PA at 70% VO_2 peak	5 times a week, 6 months	↑α diversity, ↓β diversity	[37]
Observational<br>Canada		Low (14)<br>25.5±3.3	Cycle with continuous		CRF associated with ↑α diversity, *Clostridiales, Lachnospiraceae, Erysipelotrichaceae, Coprococcus, Roseburia, Adlercreutzia*	[38]

		25.5±3.9<br>Average (12)<br>24.3±3.7<br>23.5±0.5<br>High (13)<br>26.2±5.5<br>22.8±5.5	incremental ramp starting at 50 W and increased by 30 W/min			
Observational Finland		Low Premenopausal (24, F)<br>40.4 (95% CI: 36.9–44)<br>31.7 (95% CI: 30.2–33.1)<br>Moderate Premenopausal (23, F)<br>39.7 (95% CI: 35.5–43.8)<br>27.9 (95% CI: 26.7–29.1)<br>High Premenopausal (24, F)<br>30.6 (95% CI: 25.6–35.6)<br>24.6 (95% CI: 23–26.2)	Cycle test with power increased 25 W each 2 min ending when participant reached self-declared exhaustion		Low/Moderate CRF-↑*Eubacterium rectale-Clostridium coccoides*<br>High CRF-↓*Bacteriodetes-Bacteroides*	[39]
Observational US		Healthy subjects (37, 20M/17F)<br>25.7±2.2<br>23.7±3.6	Incline increased by 2% each 2 minutes		CRF associated to ↑*Firmicutes/Bacteroidetes* ratio	[40]

Table S3: Studies investigating effects on gut microbiome based on Resistance exercise.

Study design and country	Participant characteristics Sample size and sex (n,	Intervention	Duration	Effects	References

	**M/F)**<br>**Age (mean ± SD or median [IQR], years)**<br>**BMI (mean ± SD or median [IQR], kg/m^2)**					
	**Control**	**Intervention**				
**Interventional US**		**Resistance (28, 17F/11M)**<br>**21.28±3.85**<br>**23.77±4.15**<br>**Aerobic (28, 7M/21F)**<br>**20.54±1.93**<br>**24.41±4.2**	**Resistance: 1 hour full/lower/upper body at 70–85% 1RM**<br>**Aerobic: 1 hour (2-day group cycling, 1-day rotating CRE activity) 60–90% HRmax**	**3 times a week, 2 months**	**Aerobic exercise had changes in weeks 2,3.**<br>**High 3RM squats:**<br>**↑*Ruminococcus, Lachnospiraceae, Turicibacter, Clostridium***<br>**Low 3RM squats:**<br>**↑*Siccibacter, Bacteroides, Bacteroides B, Alistipes, Oscillibacter***	**[41]**
**Clinical trial Japan**		**Healthy elderly women (29, F)**<br>**70 [66–75]**<br>**21.4 [18.8–23.1]**	**Resistance: 1-hour upper body strengthening trunk muscle arching, swaying, plank, pelvic rotation in the supine position, bird dog**<br>**Aerobic: 1 hour walking ≥3 METs**	**Weekly for resistance group & Daily for aerobic group, 3 months**	**↑*F. prausnitzii***<br>**↓*Parasutterella excrementihominis, Bilophila wadsworthia***	**[42]**

Table S4: Studies investigating effects on gut microbiome based on Aerobic exercise (AE).

**Study design and country**	**Participant characteristics**<br>**Sample size and sex (n, M/F)**<br>**Age (mean ± SD or median [IQR], years)**<br>**BMI (mean ± SD or median [IQR], kg/m^2)**		**Intervention**	**Duration**	**Effects**	**References**
	**Control**	**Intervention**				
Interventional US		Lean (18, 9M/9F)<br>25<br>25.1±6.52<br>Obese (14, 3M/11F)<br>31<br>31.14±8.57	Moderate-high intensity cycling/treadmill	30-60' 3 times a week ,6 weeks	LEAN: ↑*Faecalibacterium, Lachnospira*; ↓*Bacteroides*<br>OBESE: ↑*Bacteroides, Colinsella*; ↓*Faecalibacterium*<br>Post training: ↑*Clostridiales, Roseburia, Lachnospira, Faecalibacterium, Lachnospiraceae*, Acetate, Butyrate	[43]
Interventional		Sedentary women (17, F)	Low-moderate cycling	30-60' 3	↑β diversity	[44]

Finland		36.8±3.9<br>31.8±4.4		times a week, 6 weeks	↑*Dorea, Anaerofilum, Verrucomicrobia-Verrucomicrobiaceae Akkermansia*<br>↓*Proteobacteria (Porphyromonadaceae, Odoribacter, Desulfovibrionaceae, Enterobacteriaceae)*	
Observational<br>China		Amateur athletes (20, 16M/4F)<br>31.3±6.1<br>22.6±2.1	Marathon 21.1 km		↑*Actinobacteria-Coriobacteria-Coriobacteriales-Coriobacteriaceae, Succinivibrionaceae-Actinobacillus, Pseudobutyrivibrio, Collinsella, Mitsuokella, Ruminiclostridium-Coprococcus, Ruminococcus bicirculans, Collinsella aerofaciens, Romboutsia*;<br>↓*Roseburia*	[45]
RCT<br>Japan	Controls (33, M)<br>62-76<br>22.9 ± 2.5	Exercise group (33, M)<br>62–76<br>22.9 ± 2.5	Aerobic exercise with incremental intensity	30-45’ 3 times a week, 5 weeks	↑*Oscillospira*, ↓*C. difficile*	[46]
RCT<br>Brazil	Controls (12, M)<br>25.5±4.66<br>23.68±4.66	Healthy previously sedentary subjects (12, M)<br>25.58±5.07<br>25.28±4.11	Aerobic exercise at steady speed 60 rpm and weekly progressive overload at 60/65% VO_2 peak	50’ 3 times a week, 10 weeks	CRF associated with ↑α diversity, *Roseburia, Odoribacter, Sutterella*	[47]
Interventional<br>US	Healthy controls (10, 2M/8F)<br>46.5±13<br>23.9±4.3	Chronic Fatigue Syndrome patietns (10, 2M/8F)<br>46.5±10.5<br>24.6±3.3	Cycle test at 60-70 rpm, power increased 5 W every 20s.	Average 11.7±2.8’	↑*Bacteroidetes*	[48]
RCT<br>Finland		Prediabetic/T2D, Sedentary (18, 15M/5F)<br>49±4<br>30.5±3	SIT: HIIT 30″ exercise bouts (4-6) cycling, 4′ recovery<br>MICT: 40–60′ cycling at 60% VO_2max	3 times a week ,2 weeks	↑*Bacteroidetes*<br>↓*Firmicutes/Bacteroidetes* ratio, *Clostridium, Blautia*	[49]
RCT<br>Iran	Waitlist control participants (9, F)<br>26.37 ± 1.68<br>28.41 ± 2.81	Inactive participants (9, F)<br>23.87 ± 3.13<br>27.76 ± 1.6	Aerobic exercise 55-75% HRR	30-45’ 3 times a week, 2 weeks	↑*Lactobacillus, Bifidobacterium*	[50]
Interventional<br>Netherlands		Inactive participants (14, 7M/7F)<br>51±11<br>34.9±4.9	Exercise 65-85% HRR	2-4 times a week, 2 months	↑*Ruminococcus gauvreauii*, *Lachnospiraceae*, *Anaerostipes*<br>↑CRF associated with *Ruminococcus gauvreaui*	[51]

Longitudinal<br>Italy		Healthy college students (17, M)<br>22±2<br>22.3±2.7	Cycle, HIIT for 20% of session+LIT	55-70' 3-5 times, 9 weeks	↑*Firmicutes/Bacteroidetes* ratio, *Actinobacteria, Blautia, Bifidobacterium, Ruminococcus*<br>↓*Proteobacteria*	[52]
RCT<br>Canada	Waitlist Control (21, 2M/19F)<br>46.2±10.2<br>28.7±6.1	Celiac disease, Inactive (20, 4M/16F)<br>42±12.3<br>28.7±6.1	HIIT 90% HR_{max} by stationary bikes, ellipticals, treadmills, BW exercises	60' 2 times a week, 3 months	↑*Parabacteroides, Defluviitaleaceae*<br>↓*Roseburia, Klebsiella, Adlercreutzia*	[53]
RCT<br>China	Current PA (29, 7M/22F)<br>60±3.4<br>27.1±2.9	NAFLD, Prediabetes, AE (29, 6M/23F)<br>59±4.4<br>27.3±3.6<br>NAFLD, Prediabetes, AE+Diet (29, 7M/22F)<br>60±3.5<br>26.4±2.9	Nordic brisk walking+stretching+other group exercises 60-75% VO_2max	30-60' 2-3 times a week, average 37 weeks	↑α Index, *Bilophila, Erysipelotrichaceae*, *Hungatella, Lachnospiraceae*, *Roseburia*	[54]
RCT<br>China		Low carbohydrate+HIIT (13, F)<br>21.4±2.9<br>24.8±2<br>Low carbohydrate+MICT (12, F)<br>21.8±3.1<br>24.8±1.9	HIIT: Cycling+sprinting for 6s against resistance+9s rest, 10 times<br>MICT 30' cycling 50-60% of VO_2max	1 month	LC-HIIT: ↓*Bifidobacterium, Alistipes*<br>LC-MICT: ↑*Blautia*, ↓*Alistipes*	[55]
Interventional<br>Japan		Ultramarathon runners (9, M)<br>46.9±5.8<br>21.6±1.1	Marathon 96.102-99.12 km	38-44 hours	↑*Collinsella aerofaciens, Catenibacillus scindens, Clostridium*, *Alistipes putredinis*<br>↓*Blautia luti, Eubacterium rectale, Faecalibacterium prausnitzii*	[56]
Interventional<br>China	Control group (11, M)<br>21.82±1.27<br>17.73±1.19	Experimental group (11, 4M/7F)<br>22.55±1.08<br>17.65±0.9	Jogging at 8-9km/h 3 times per week for 4–7 km	2 months	↑*Blautia, Eubacterium hallii*;<br>↓*Agathobacter*	[57]
RCT<br>China	Psychoeducation Clinically well (84, 36M/48F)<br>12.6±0.1<br>18.5±3.4<br>Sub-threshold symptoms (28,	Clinically well (84, 36M/48F)<br>12.6±0.1<br>17.9±3.5<br>Sub-threshold symptoms (28, 16M/12F)<br>12.6±0.1	Moderate intensity running	30' 4 days a week, 3 months	↑*Coprococcus, Blautia, Dorea, Tyzzerella-Tyzzerella nexilis, Ruminococcus obeum*	[58]

	8M/20F)<br>12.6±0.1<br>18.6±3.2	18.7±3.3				
RCT<br>Korea	Healthy (14)<br>30-50	Healthy (13)<br>30-50<br>NS	Aerobic exercise	30' 3 sessions per week, 3 months	↑α diversity, *Firmicutes, Tenericutes, Verrucomicrobia and Dialister succinatiphilus* ↓*Proteobacteria, Lentishaerae, Bacteroides fragilis, Phascolarctobacterium faecium, Megasphaera elsdenii*	[59]
Clinical Trial<br>Japan		College long distance runners (13, 9M/4F)<br>19-21<br>BMI: M 19.2±0.9<br>F 21.3±1.2	Training+race	4 weeks	↑α diversity, *Firmicutes*; ↓*Bacteroidetes*	[60]
Observational<br>Ireland		Athletes (3, M)<br>26.5±1.3<br>24.4±1.4	3748.91 km rowing	33 days 22 hours	↑α diversity, *Subdoligranulum*, *Dorea longicatena, Roseburia hominis*; ↓*Bacteroides finegoldii*	[61]
RCT<br>Slovakia		Swimmers (24, 17M/7F)<br>16–25<br>NS	HIIT	7 weeks	↑α diversity, *Gammaproteobacteria, Burkholderiales, Barnesiellaceae, Sutterellaceae, Butyricimonas, Alistipes, Lactococcus, Parasutterella*	[62]
RCT<br>Norway	Control (18, M)<br>19±2<br>23.6±1.8	Carbohydrate supplemented (27, M)<br>20±1<br>24.1±2.3<br>Protein supplemented (28, M)<br>20±1<br>23.3±2.1	Arctic military training exercise	4 days	↑α diversity, *Firmicutes/Bacteroidetes* ratio, *Acidaminococcus, Fusobacterium, Peptoniphilus, Peptostreptococcus, Staphylococcus*<br>↓*Bacteroides, Collinsella, Faecalibacterium, Roseburia*	[63]
Observational<br>Spain		Cross-country runners (40, M)<br>35.8±8<br>22.8±2.1	Moderate intensity treadmill, slope of 1% at 10 km/h, with increments of 0.3 km/h every 30 s	Average time to run 1 km<br>3.22±0.26 min	↑*Romboutsia, Escherichia coli*, Ruminococcaceae, *Blautia, Ruminiclostridium Clostridium phoceensis*	[64]

Table S5: Studies investigating effects on gut microbiome based on combined Resistance and Aerobic exercise.

Study design	Participant characteristics	Intervention	Duration	Effects	References

and country	Sample size and sex (n, M/F)<br>Age (mean ± SD or median [IQR], years)<br>BMI (mean ± SD or median [IQR], kg/m^2)					
	Control	Intervention				
RCT<br>China		Medication naïve with prediabetes (39, M)<br>Responders:<br>43.29±3.27<br>28.78±1.08<br>Non-responders:<br>36±4.55<br>29.82±1.75	High intensity aerobic and resistance interval training, 80–95% HR_{max}	3 times a week, 3 months	↑*Streptococcus mitis, A. muciniphila*<br>↓*Bacteroides*	[65]
RCT<br>Spain	Healthy control subjects (14)<br>7-12<br>NS	Obese pediatric patients (25)<br>7–12<br>NS	Sprint of 30” max cadence at 3′30″, 4′30″, 5′30″, 6′30″) & strength training (30–70% 1RM)	2 times a week, 3 months	↑*Blautia, Dialister, Roseburia*;<br>↓*Proteobacteria, Gammaproteobacteria*	[66]
RCT<br>China	Control group (6, F)<br>67.5±4.28<br>23.23±4.2	Exercise group (6, F)<br>69.83±4.28<br>22.69±1.21	Progressive overload aerobic, resistance exercise	1 hour 4 times a week, 2 months	↑*Prevotella, Verrucomicrobia*;<br>↓Proteobacteria	[67]
Interventional<br>US		Sedentary adults (15, 14M/1F)<br>58±8<br>27.4±6.4	20/30′ aerobic exercise+3 sets of 8 reps of resistance exercise-low intensity	50’ 3 times a week 6 months	↑ *Bifidobacterium, Oscillospira, Anaerostipes*;<br>↓*Prevotella, Succinivibrio*	[68]
Prospective<br>Ireland		Exercise (25, 11M/14F)<br>35 [28-38]<br>27.9 [25.1-29.2]	Moderate aerobic exercise minimum 18-32 min, 15% to 20%.<br>7 machine-based resistance exercise<br>3 sets of 8-12 repetitions at 70% 1RM	3 times per week, 2 months	↑α diversity	[69]
Clinical trial<br>Germany		NAFLD patients (41, 27M/14F)<br>41 (24-61)<br>31.3 [27.3-34]	ARE individualized training program at moderate intensity	3-5 sessions/week at 8 weeks	↑α diversity, *Bacteroidetes, Euryarchaeota*;<br>↓*Actinobacteria, Bifidobacterium*	[70]
RCT<br>Iran	Multiple sclerosis+Routine PA (21, 5M/16F)<br>36.38±9.13	Multiple sclerosis+Home based exercise (21, 6M/15F)<br>35.06±8.18	Combined aerobic exercise 50-75% of HRR (3 times/week) & resistance exercise with elastic bands, body weight (2	6 months	↑*Prevotella*	[71]

	22.62 ± 2	23.47 ± 2.61	times/week)			
RCT<br>Australia		T2D+Moderate intensity continuous training (7, 1M/6F)<br>62.5±5.5<br>NS<br>T2D+High-intensity interval training (5, 4M.1F)<br>64.4±8.2<br>NS	MICT: 2 sessions ARE (AE at 55- 69% HRmax+30’ RE at 11-13 RPE) & 2 sessions AE only<br>HIIT: 4’ AE at 85–95% of HRmax+8-1’ intervals of RE at 17-18 RPE	26-52.5’ 3-4 sessions/week, 8 weeks	MICT: ↑*Bifidobacterium, A. muciniphila, Lachnospira eligens, Enterococcus, Clostridium*<br>HIIT: ↑*Eryspelothrichales, Oscillospirales, Methanobrevibacter smithii*	[72]
RCT<br>Denmark	Standard care (34)	Sample population (86, 45M/41F)<br>54.3 ± 8.9<br>NS<br>Lifestyle intervention group	2-3 sessions combined with resistance training, physically active in leisure time (≥ 10,000 steps/day)	30-60’ 5-6 times a week, 1 year	↑α diversity, *Bacteroides, Roseburia*; ↓*Firmicutes/Bacteroidetes* ratio	[32,73]
Case study<br>Ireland		Unfit participants (2, M)<br>30, 33<br>28.6, 31.7	Regular AE+2 sessions/week RE	6 months	↑α diversity<br>Marathoner: ↑*Veillonella parvula*; ↓*Agathobacter rectalis*<br>Triathlete: ↑*Akkermansia muciniphila, Methanobrevibacter smithii*; ↓*Bifidobacterium animalis*	[74]

Table S6: Studies investigating gut microbiome in professional sportspersons.

**Study design and country**	**Participant characteristics<br>Sample size and sex (n, M/F)<br>Age (mean ± SD or median [IQR], years)<br>BMI (mean ± SD or median [IQR], kg/m²)**		**Sport**	**Effects**	**References**
	**Control**	**Intervention**			
Cohort study<br>Sweden	Community dwelling (70, 37M/33F)<br>72 [69-76]<br>NS	Senior orienteers (28, 16M/12F)<br>68.5 [67-72]<br>NS	Orienteering	↑*F. prausnitzii*<br>↓*Parasutterella excrementihominis, Bilophila wadsworthia*	[75]

Comparative<br>China	Lower-level group (16, 8M/8F)<br>20.19±1.22<br>21.72±1.21	Higher level group (12, 5M/7F)<br>20.08±1.83<br>22.24±2.12	Martial arts	↑α diversity, *Parabacteroides, Phascolarctobacterium, Oscillibacter, Bilophila*	[76]
Observational<br>US		Cyclists (33, 22M/11F)<br>33 (19–49)<br>NS	Cycling	↑*Methanobrevibacter smithii, Prevotella*	[77]
Case-control<br>Japan	Healthy, non-athletic (14. F)<br>20.9±0.3<br>21.3±0.6	Endurance Runners (15, F)<br>20.5±1.2<br>17.8±0.2	Athletics	↑*Faecalibacterium, Haemophilus, Rothia, Ruminococcus gnavus*	[78]
Cross-sectional<br>Austria	Anorexia Nervosa pts. (18, F)<br>22.44±3.2<br>15.29±1.28<br>Normal weight (26, F)<br>24.93±3.75<br>21.89±1.73<br>Athletes (20, F)<br>25.52±3.98<br>26.99±1.13	Athletes (20, F)<br>22.15±3.86<br>22.14±1.76	Athletics	↑α diversity	[79]
Observational<br>Ireland		Sports Athletes (37, 23M/14F)<br>27±5<br>NS	16 sports	High dynamic component: ↑compositionally distinct GM.<br>High dynamic+static component: ↑functionally distinct GM<br>Moderate dynamic component (Fencing): ↑*Streptococcus suis*, *Clostridium bolteae*, *Lactobacillus, Anaerostipes hadrus*<br>High dynamic+low static component (Field hockey): ↑*Bifidobacterium animalis*, *Lactobacillus acidophilus*, *Prevotella intermedia, F. prausnitzii*<br>High dynamic+static component (Rowing): ↑*Bacteroides caccae*	[80]
Cohort study<br>Slovakia	Healthy controls<br>64.9 (62.1-67.7)<br>27.3 (24.9-29.7)	Endurance athletes (13, M)<br>63.5 (61.4-65.7)<br>24.8 (24-25.6)	Athletics	↑*Prevotella, Bacteroidetes*	[81]
Observational<br>China	Non-elite ethletes (7, F)<br>19<br>NS	Elite athletes (12, F)<br>19<br>NS	Rowing	↑α, β diversity, *Firmicutes/Bacteroidetes* ratio, *Firmicutes (Clostridiales*, *Ruminococcaceae*, *Faecalibacterium*), *Proteobacteria, Lachnospiraceae*<br>↓*Bacteriodetes, Pervotella*	[82]
Observational<br>Korea	No regular exercise habits (15)	Bodybuilders (15, M)	Bodybuilding, Athletics	Bodybuilders-↑*Firmicutes (Faecalibacterium, Clostridium, Eisenbergiella), Proteobacteria (Sutterella, Haemophilus)*	[83]

	26.3±2<br>25.9±4.2	24.9±2.7<br>28.1±2.6<br>Distance runners (15, M)<br>19.8±0.8<br>20.5±0.9		↓*Bacteroides stercoris, Parasutterella, Proteobacteria-Acinetobacter, Bifidobacterium adolescentis/longum, Lactobacillus sake, Blautia wexlerae, Eubacterium hallii*<br>Runners-↓*Firmicutes (Blautia), Leuconostoc, Weissella, Bacteroides caccae*	
Observational Poland	Sedentary (46, 14M/31F)<br>14-72<br>NS	Skiers, Athletes (71)<br>Skiers (F)<br>34.5±3.5<br>Athletes (F)<br>28.6±4.6<br>Skiers (M)<br>8.6±4.6<br>Athletes (M)<br>21±3.3<br>BMI NS	Skiing, Athletics	↑GM Diversity, *Bacteroides, Prevotella*	[84]
Matching China	Active Adults (22, 12M/10F)<br>21.27±2.47<br>22.28±2.71<br>Inactive Adults (22, 12M/10F)<br>21.27±2.51<br>21.89±2.81	Elite Athletes (22, 12M/10F)<br>21.55±2.42<br>20.67±5.26	Athletics	↑*Clostridiaceae, Megamonas_rupellensis, Bilophila, Faecalicoccus*	[85]
Observational US		163km marathon runner (1, M)<br>32<br>22.1	Atheltics	↑α diversity, *Firmicutes/Bacteroidetes* ratio<br>↑*Veillonella, Streptococcus concomitant, Faecalibacterium, Haemophilus*<br>↓*Alloprevotella, Subdolingranulum*	[86]
Case-control Finland	Active (27, 14M/13F)<br>27.4±5.6<br>24±3.5	Skiers (27, 14M/13F)<br>27.1±5.1<br>22.05±1.8	Skiing	↑α diversity, *Eubacterium eligens*; ↓*Phascolarctobacterium, Lachnospiraceae, Bacteroides, Lachnoclostridium, Akkermansia*<br>Amount of PA associated with ↑*Pasteurellaceae*, ↓*Acidamidococcaceae*<br>Training load associated with ↓*Enterobacteriaceae, Bacteroidaceae, Veillonellaceae*	[87]

Table S7. Studies investigating effects of probiotic supplementation in sports performance.

**Study design and country**	**Participant characteristics**<br>**Sample size and sex (n, M/F)**<br>**Age (mean ± SD or median [IQR], years)**	**Intervention**	**Dose, frequency, Duration**	**Effects**	**References**

	BMI (mean ± SD or median [IQR], kg/m^2)					
	**Control**	**Intervention**				
RCT<br>Australia	Elite rugby union players (10, M)<br>26.6±2.9<br>20.7±1.7	Elite rugby union players (9, M)<br>27.0±3.2<br>20.6±1.7	*Lactobacillus acidophilus, Bifidobacterium animalis, B. bifidum, B. lactis, Streptococcus thermophilus,* Sccharomyces *boulardii*	60 billion bacteria, 250 mg yeast	Leg heaviness, muscle soreness lower	[88]
RCT<br>Poland	Professional road cyclists (M)<br>23.25 years<br>20.80 ±1.11	Professional road cyclists (M)<br>21.28 years<br>21.79 ±1.90	*B. bifidum, B. breve, B, infantis, B. longum, L. acidophilus, L. bulgaricus, L. casei, L. fermentum, L. helveticus, L. plantarum, L. rhamnosus, S. thermophilus, Lactococcus lactis,*	1 × 10^{11} CFU daily for 16 weeks	Increased aerobic capacity, maximal oxygen uptake, duration of exercise to failure, load on the ergometer. decrease in heart rates, feeling of less discomfort during the exercise test	[89]
RCT<br>Israel	Elite cyclists (16, M)<br>29.5±6.2<br>23.5±1.9	Elite cyclists (11, M)<br>25.9±4.6 years<br>22.6±2.7	*B. animalis*, *B. longum, Bacillus subtilis, Enterococcus faecium, L. helveticus*	15 billion CFU for 90 days	Lower incidence of nausea, belching, and vomiting at rest, and decreased incidence of GI symptoms during training	[90]
RCT<br>Austria	Trained volunteer athletes (15, 10/5)<br>26.6 ± 3.5 years<br>21.2 ± 2.7	Trained volunteer athletes (14, 6/8)<br>25.7 ± 3.5 years<br>22.2 ± 1.5	*B. bifidum*, *B. lactis*, *E. faecium*, *L. acidophilus*, *L. brevis*, *Lactococcus lactis*	1 × 10^{10} CFU/day for 12 weeks	Ratio of subjects in. control group who had one or more URTI symptoms increased	[91]

RCT<br>USA	Division I Female Athletes (12, F)<br>19.6 ± 1.0 years (	Division I Female Athletes (11, F)<br>19.6 ± 1.0 years	*B. subtilis*	Once a day for 10 weeks-5 billion CFU	Improved squat 1RM, deadlift 1RM, bench press 1RM, vertical jump, RF MT, and Body Fat %. Of these, a significant group × time interaction was noted for BF% where greater reductions were observed.	[92]
RCT<br>Canada	Elite athletes (19, 4/15)<br>22.8 ± 2.5 years<br>22.9 ± 2.2	Elite athletes (20, 5/15)<br>23.5 ± 2.7 years<br>22.9 ± 2.2	*L. helveticus*	2×10^{10} CFU daily for 14 weeks.	Shortened URTI episode duration, decreased number of symptoms, sense of vigor increased	[93]
RCT<br>Japan	Athletes (24, M)<br>20.5±0.8	Athletes (26, M)<br>20.8±0.8	*Lactococcus lactis*	100 billion cells for 13 days	Increased CD86, decreased cumulative days of fatigue	[94]
RCT<br>Austria	Endurance Trained men-triathletes, runners, cyclists (11, M)<br>37.6 ± 4.7<br>23.7 ± 2.2	Endurance Trained men-triathletes, runners, cyclists (12, M)<br>38.2 ± 4.4<br>23.9 ± 3.1	*B. bifidum, B. lactis, E. faecium, L. acidophilus, L. brevis, Lactococcus lactis*	10^{10} CFU/day 14 weeks	Zonulin decrease 25% in feaces TNFL-6, protein oxidation decreased	[95]
RCT<br>Italy	Amateur male athletes (12, M)<br>32.03 ± 6.12 years	Amateur male athletes (12, M)<br>32.03 ± 6.12 years	*L. paracasei,L. rhamnosus*	10^9 cells/day 4 weeks	increased plasma antioxidant levels, neutralized ROS	[96]
Single-blind, Crossover<br>USA		recreationally trained males<br>(29, M)<br>21.5 ± 2.8 years	*Bacillus coagulans*+casein	1×10^9 CFU/day+20 g casein for 14 days	increased perceived recovery status, reduced muscle	[97]

					soreness.	
RCT<br>England		(30, 5/25)<br>35 ± 1 years	*B. animalis, B. bifidum, L. acidophilus*	daily dose 30 $\times 10^9$ CFU<br>12 weeks before and 6 days after triathlon	Reduced post-race endotoxin levels, non-significant faster times for swim and cycle stage	[98]
RCT<br>Iran	endurance swimmers girls<br>(23, F)<br>13.8 ±1.8	endurance swimmers girls<br>(23, F)<br>13.8 ±1.8	*B. bifidum, L. acidophilus, L. delbrueckii, S. salivarus*	4×10^{10} cfu/ml-400 ml	Improvement in VO2max, reduced respiratory infection	[99]
RCT<br>Malaysia	Badminton player<br>15<br>19.9 ± 1.3	Badminton players<br>15<br>19.5 ± 1.0	*L. casei*+orange juice	3×10^{10} CFU daily for 6 weeks	improved aerobic capacity	[100]
RCT<br>Australia	Male runner<br>(5, M)<br>27 ± 2 years	Male runner<br>(5, M)<br>27 ± 2 years	*B. bifidum, B. breve, B. lactis, L. acidophilus, L. casei, L. fermentum, L. plantarum, L. rhamnosus, S. thermophilus*	4 weeks-45 billion CFU	increased run time to fatigue	[101]
RCT matched pair<br>England	Marathon Runners<br>(12, 2/10)<br>36.1 ± 7.5	Marathon Runners<br>(12, 2/10)<br>34.8 ± 6.9	*B. animalis, B. bifidum, L. acidophilus*	2.5×10^{10} CFU/day for 28 days	Lower GI symptoms, placebo had more reduction in speed during last third of race	[102]
RCT Australia	Competitive Cyclists<br>(47, 18/29)<br>M- 35.2 ± 10.3<br>F- 36.5 ± 8.6	Competitive Cyclists<br>(50, 17/33)<br>M- 36.4 ± 8.9<br>F- 35.6 ± 10.2	*L. fermentum*	1×10^9 cell/day<br>for 11 weeks	In males lower respiratory illness, GI symptoms at high training loads, cold and flu medication-greater uptake, increase mild GI symptom, reduction in exercise induced cytokine	[103]
Double Blind Experimental	STUDY 1:<br>Triathletes	Study 1:<br>Triathletes	*L. plantarum*	2 capsules per day (3×10^{10}	Alleviate oxidative stress	[104]

Taiwan	9<br>21.1 ± 1.5<br>Study 2:<br>8<br>20.1 ± 0.3	9<br>20.2 ± 0.7<br>Study 2:<br>8<br>22.3 ± 1.2		CFU/day)<br>Study 1-4 weeks<br>Study 2-3 weeks	creatine kinase, Thioredoxin, and Myeloperoxidase indices after a marathon<br>Decreased TNF-α, IL-6, IL-8<br>Increase in branched amino acids<br>Increased IL-10	

* Studies with multiple modes of exercise are bolded; All participants are healthy, morbidities are mentioned wherever applicable; ↓ decrease; ↑ increase; Abbreviations: BMI, body mass index; F, female; M, male; CFU, Colony Forming Unit; NS, not specified; RCT, Randomized controlled trial; SIT, Sprint Interval Training; HIIT, High Intensity Interval Training; MICT, Moderate Intensity Continuous Training

GM & PA

References

1. Mika, A.; Van Treuren, W.; González, A.; Herrera, J.J.; Knight, R.; Fleshner, M. Exercise is More Effective at Altering Gut Microbial Composition and Producing Stable Changes in Lean Mass in Juvenile versus Adult Male F344 Rats. *PloS one* **2015**, *10*, e0125889-e0125889, doi:10.1371/journal.pone.0125889.
2. Matsumoto, M.; Inoue, R.; Tsukahara, T.; Ushida, K.; Chiji, H.; Matsubara, N.; Hara, H. Voluntary Running Exercise Alters Microbiota Composition and Increases n-Butyrate Concentration in the Rat Cecum. *Bioscience, biotechnology, and biochemistry* **2008**, *72*, 572-576, doi:10.1271/bbb.70474.
3. Górniak, W.; Cholewińska, P.; Szeligowska, N.; Wołoszyńska, M.; Soroko, M.; Czyż, K. Effect of Intense Exercise on the Level of Bacteroidetes and Firmicutes Phyla in the Digestive System of Thoroughbred Racehorses. *Animals (Basel)* **2021**, *11*, 290, doi:10.3390/ani11020290.
4. Evans, C.C.; LePard, K.J.; Kwak, J.W.; Stancukas, M.C.; Laskowski, S.; Dougherty, J.; Moulton, L.; Glawe, A.; Wang, Y.; Leone, V., et al. Exercise prevents weight gain and alters the gut microbiota in a mouse model of high fat diet-induced obesity. *PloS one* **2014**, *9*, e92193-e92193, doi:10.1371/journal.pone.0092193.
5. Choi, J.J.; Eum, S.Y.; Rampersaud, E.; Daunert, S.; Abreu, M.T.; Toborek, M. Exercise attenuates PCB-induced changes in the mouse gut microbiome. *Environmental health perspectives* **2013**, *121*, 725-730, doi:10.1289/ehp.1306534.
6. Campbell, S.C.; Wisniewski, P.J.; Noji, M.; McGuinness, L.R.; Häggblom, M.M.; Lightfoot, S.A.; Joseph, L.B.; Kerkhof, L.J. The Effect of Diet and Exercise on Intestinal Integrity and Microbial Diversity in Mice. *PloS one* **2016**, *11*, e0150502, doi:10.1371/journal.pone.0150502.
7. Castro, A.P.; Silva, K.K.S.; Medeiros, C.S.A.; Alves, F.; Araujo, R.C.; Almeida, J.A. Effects of 12 weeks of resistance training on rat gut microbiota composition. *Journal of experimental biology* **2021**, *224*, doi:10.1242/jeb.242543.
8. Chen, H.; Shen, L.; Liu, Y.; Ma, X.; Long, L.; Ma, X.; Ma, L.; Chen, Z.; Lin, X.; Si, L., et al. Strength Exercise Confers Protection in Central Nervous System Autoimmunity by Altering the Gut Microbiota. *Frontiers in immunology* **2021**, *12*, 628629-628629, doi:10.3389/fimmu.2021.628629.
9. Giacco, A.; delli Paoli, G.; Simiele, R.; Caterino, M.; Ruoppolo, M.; Bloch, W.; Kraaij, R.; Uitterlinden, A.G.; Santillo, A.; Senese, R., et al. Exercise with food withdrawal at thermoneutrality impacts fuel use, the microbiome, AMPK phosphorylation, muscle fibers, and thyroid hormone levels in rats. *Physiological reports* **2020**, *8*, e14354-n/a, doi:10.14814/phy2.14354.
10. Meng, Y.; Chen, L.; Lin, W.; Wang, H.; Xu, G.; Weng, X. Exercise Reverses the Alterations in Gut Microbiota Upon Cold Exposure and Promotes Cold-Induced Weight Loss. *Frontiers in physiology* **2020**, *11*, 311-311, doi:10.3389/fphys.2020.00311.
11. Lamoureux, E.V.; Grandy, S.A.; Langille, M.G.I. Moderate Exercise Has Limited but Distinguishable Effects on the Mouse Microbiome. *mSystems* **2017**, *2*, doi:10.1128/mSystems.00006-17.
12. Ribeiro, F.M.; Ribeiro, C.F.A.; G, A.C.M.; Castro, A.P.; Almeida, J.A.; Franco, O.L.; Petriz, B.A. Limited Effects of Low-to-Moderate Aerobic Exercise on the Gut Microbiota of Mice Subjected to a High-Fat Diet. *Nutrients* **2019**, *11*, 149, doi:10.3390/nu11010149.
13. Almeida, M.L.M.d.; Feringer, J.W.H.; Carvalho, J.R.G.; Rodrigues, I.M.; Jordão, L.R.; Fonseca, M.G.; Carneiro de Rezende, A.S.; de Queiroz Neto, A.; Weese, J.S.; Costa, M.C.d., et al. Intense Exercise and Aerobic Conditioning Associated with Chromium or L-Carnitine Supplementation Modified the Fecal Microbiota of Fillies. *PloS one* **2016**, *11*, e0167108-e0167108, doi:10.1371/journal.pone.0167108.
14. Houghton, D.; Stewart, C.J.; Stamp, C.; Nelson, A.; Aj Ami, N.J.; Petrosino, J.F.; Wipat, A.; Trenell, M.I.; Turnbull, D.M.; Greaves, L.C. Impact of Age-Related Mitochondrial Dysfunction and Exercise on Intestinal Microbiota Composition. *The journals of gerontology. Series A, Biological sciences and medical sciences* **2018**, *73*, 571-578, doi:10.1093/gerona/glx197.
15. Walshe, N.; Cabrera-Rubio, R.; Collins, R.; Puggioni, A.; Gath, V.; Crispie, F.; Cotter, P.D.; Brennan, L.; Mulcahy, G.; Duggan, V. A Multiomic Approach to Investigate the Effects of a Weight Loss Program on the Intestinal Health of Overweight Horses. *Frontiers in veterinary science* **2021**, *8*, 668120-668120, doi:10.3389/fvets.2021.668120.
16. Lambert, J.E.; Myslicki, J.P.; Bomhof, M.R.; Belke, D.D.; Shearer, J.; Reimer, R.A. Exercise training modifies gut microbiota in normal and diabetic mice. *Applied physiology, nutrition, and metabolism* **2015**, *40*, 749-752, doi:10.1139/apnm-2014-0452.
17. Bressa, C.; Bailén-Andrino, M.; Pérez-Santiago, J.; González-Soltero, R.; Pérez, M.; Montalvo-Lominchar, M.G.; Maté-Muñoz, J.L.; Domínguez, R.; Moreno, D.; Larrosa, M. Differences in gut microbiota profile

between women with active lifestyle and sedentary women. *PLoS ONE* **2017**, *12*, doi:10.1371/journal.pone.0171352.
18. Langsetmo, L.; Johnson, A.; Demmer, R.; Fino, N.; Orwoll, E.; Ensrud, K.; Hoffman, A.R.; Cauley, J.A.; Shmagel, A.; Meyer, K., et al. The Association between Objectively Measured Physical Activity and the Gut Microbiome among Older Community Dwelling Men. *The journal of nutrition, health & aging* **2019**, *23*, doi:10.1007/s12603-019-1194-x.
19. Castellanos, N.; Diez, G.G.; Antúnez-Almagro, C.; Bailén, M.; Bressa, C.; Soltero, R.G.; Pérez, M.; Larrosa, M. A Critical Mutualism – Competition Interplay Underlies the Loss of Microbial Diversity in Sedentary Lifestyle. *Frontiers in Microbiology* **2019**, *10*, doi:10.3389/fmicb.2019.03142.
20. Paulsen, J.A.; Ptacek, T.S.; Carter, S.J.; Liu, N.; Kumar, R.; Hyndman, L.; Lefkowitz, E.J.; Morrow, C.D.; Rogers, L.Q. Gut microbiota composition associated with alterations in cardiorespiratory fitness and psychosocial outcomes among breast cancer survivors. *Supportive care in cancer : official journal of the Multinational Association of Supportive Care in Cancer* **2017**, *25*, doi:10.1007/s00520-016-3568-5.
21. Gallè, F.; Valeriani, F.; Cattaruzza, M.S.; Gianfranceschi, G.; Liguori, R.; Antinozzi, M.; Mederer, B.; Liguori, G.; Spica, V.R. Mediterranean Diet, Physical Activity and Gut Microbiome Composition: A Cross-Sectional Study among Healthy Young Italian Adults. *Nutrients* **2020**, *12*, doi:10.3390/nu12072164.
22. Manor, O.; Dai, C.L.; Kornilov, S.A.; Smith, B.; Price, N.D.; Lovejoy, J.C.; Gibbons, S.M.; Magis, A.T. Health and disease markers correlate with gut microbiome composition across thousands of people. *Nature Communications* **2020**, *11*, doi:10.1038/s41467-020-18871-1.
23. Whisner, C.M.; Maldonado, J.; Dente, B.; Krajmalnik-Brown, R.; Bruening, M. Diet, physical activity and screen time but not body mass index are associated with the gut microbiome of a diverse cohort of college students living in university housing: a cross-sectional study. *BMC Microbiol* **2018**, *18*, 210, doi:10.1186/s12866-018-1362-x.
24. Castro-Mejía, J.L.; Khakimov, B.; Krych, Ł.; Bülow, J.; Bechshøft, R.L.; Højfeldt, G.; Mertz, K.H.; Garne, E.S.; Schacht, S.R.; Ahmad, H.F., et al. Physical fitness in community-dwelling older adults is linked to dietary intake, gut microbiota, and metabolomic signatures. *Aging Cell* **2020**, *19*, e13105, doi:10.1111/acel.13105.
25. Zhu, Q.; Jiang, S.; Du, G. Effects of exercise frequency on the gut microbiota in elderly individuals. *MicrobiologyOpen* **2020**, *9*, doi:10.1002/mbo3.1053.
26. Bai, J.; Hu, Y.; Bruner, D.W. Composition of gut microbiota and its association with body mass index and lifestyle factors in a cohort of 7–18 years old children from the American Gut Project. *Pediatric Obesity* **2019**, *14*, doi:10.1111/ijpo.12480.
27. Zhang, W.; Li, J.; Lu, S.; Han, N.; Miao, J.; Zhang, T.; Qiang, Y.; Kong, Y.; Wang, H.; Gao, T., et al. Gut microbiota community characteristics and disease-related microorganism pattern in a population of healthy Chinese people. *Scientific Reports* **2019**, *9*, doi:10.1038/s41598-018-36318-y.
28. Carter, S.J.; Hunter, G.R.; Blackston, J.W.; Liu, N.; Lefkowitz, E.J.; Pol, W.J.V.D.; Morrow, C.D.; Paulsen, J.A.; Rogers, L.Q. Gut microbiota diversity associates with cardiorespiratory fitness in post-primary treatment breast cancer survivors. *Experimental physiology* **2019**, *104*, doi:10.1113/EP087404.
29. Lin, T.-Y.; Wu, P.-H.; Lin, Y.-T.; Hung, S.-C. Characterization of Gut Microbiota Composition in Hemodialysis Patients With Normal Weight Obesity. *The Journal of Clinical Endocrinology & Metabolism* **2020**, *105*, doi:10.1210/clinem/dgaa166.
30. Palmas, V.; Pisanu, S.; Madau, V.; Casula, E.; Deledda, A.; Cusano, R.; Uva, P.; Vascellari, S.; Loviselli, A.; Manzin, A., et al. Gut microbiota markers associated with obesity and overweight in Italian adults. *Scientific Reports* **2021**, *11*, doi:10.1038/s41598-021-84928-w.
31. Houttu, V.; Boulund, U.; Nicolaou, M.; Holleboom, A.G.; Grefhorst, A.; Galenkamp, H.; Born, B.-J.v.d.; Zwinderman, K.; Nieuwdorp, M. Physical Activity and Dietary Composition Relate to Differences in Gut Microbial Patterns in a Multi-Ethnic Cohort—The HELIUS Study. *Metabolites* **2021**, *11*, doi:10.3390/metabo11120858.
32. Walker, R.L.; Vlamakis, H.; Lee, J.W.J.; Besse, L.A.; Xanthakis, V.; Vasan, R.S.; Shaw, S.Y.; Xavier, R.J. Population study of the gut microbiome: associations with diet, lifestyle, and cardiometabolic disease. *Genome Medicine* **2021**, *13*, doi:10.1186/s13073-021-01007-5.
33. Santarossa, S.; Sitarik, A.R.; Johnson, C.C.; Li, J.; Lynch, S.V.; Ownby, D.R.; Ramirez, A.; Yong, G.L.; Cassidy-Bushrow, A.E. Associations of physical activity with gut microbiota in pre-adolescent children. *Physical Activity and Nutrition* **2021**, *25*, doi:10.20463/pan.2021.0023.
34. Magzal, F.; Shochat, T.; Haimov, I.; Tamir, S.; Asraf, K.; Tuchner-Arieli, M.; Even, C.; Agmon, M. Increased physical activity improves gut microbiota composition and reduces short-chain fatty acid

concentrations in older adults with insomnia. *Scientific Reports* **2022**, *12*, doi:10.1038/s41598-022-05099-w.
35. Shivani, S.; Kao, C.-Y.; Chattopadhyay, A.; Chen, J.-W.; Lai, L.-C.; Lin, W.-H.; Lu, T.-P.; Huang, I.-H.; Tsai, M.-H.; Teng, C.-H., et al. Uremic Toxin-Producing Bacteroides Species Prevail in the Gut Microbiota of Taiwanese CKD Patients: An Analysis Using the New Taiwan Microbiome Baseline. *Frontiers in Cellular and Infection Microbiology* **2022**, *12*, doi:10.3389/fcimb.2022.726256.
36. Frugé, A.D.; Smith, K.S.; Bail, J.R.; Rais-Bahrami, S.; Demark-Wahnefried, W. Biomarkers Associated With Tumor Ki67 and Cathepsin L Gene Expression in Prostate Cancer Patients Participating in a Presurgical Weight Loss Trial. *Frontiers in Oncology* **2020**, *10*, doi:10.3389/fonc.2020.544201.
37. Kern, T.; Blond, M.B.; Hansen, T.H.; Rosenkilde, M.; Quist, J.S.; Gram, A.S.; Ekstrøm, C.T.; Hansen, T.; Stallknecht, B.; Kern, T., et al. Structured exercise alters the gut microbiota in humans with overweight and obesity—A randomized controlled trial. *International Journal of Obesity 2019 44:1* **2019**, *44*, doi:10.1038/s41366-019-0440-y.
38. Estaki, M.; Pither, J.; Baumeister, P.; Little, J.P.; Gill, S.K.; Ghosh, S.; Ahmadi-Vand, Z.; Marsden, K.R.; Gibson, D.L. Cardiorespiratory fitness as a predictor of intestinal microbial diversity and distinct metagenomic functions. *Microbiome* **2016**, *4*, doi:10.1186/s40168-016-0189-7.
39. Yang, Y.; Shi, Y.; Wiklund, P.; Tan, X.; Wu, N.; Zhang, X.; Tikkanen, O.; Zhang, C.; Munukka, E.; Cheng, S. The Association between Cardiorespiratory Fitness and Gut Microbiota Composition in Premenopausal Women. *Nutrients* **2017**, *9*, doi:10.3390/nu9080792.
40. Durk, R.P.; Castillo, E.; Márquez-Magaña, L.; Grosicki, G.J.; Bolter, N.D.; Lee, C.M.; Bagley, J.R. Gut Microbiota Composition is Related to Cardiorespiratory Fitness in Healthy Young Adults. *International journal of sport nutrition and exercise metabolism* **2019**, *29*, doi:10.1123/ijsnem.2018-0024.
41. Bycura, D.; Santos, A.C.; Shiffer, A.; Kyman, S.; Winfree, K.; Sutliffe, J.; Pearson, T.; Sonderegger, D.; Cope, E.; Caporaso, J.G. Impact of Different Exercise Modalities on the Human Gut Microbiome. *Sports* **2021**, *9*, doi:10.3390/sports9020014.
42. Morita, E.; Yokoyama, H.; Imai, D.; Takeda, R.; Ota, A.; Kawai, E.; Hisada, T.; Emoto, M.; Suzuki, Y.; Okazaki, K. Aerobic Exercise Training with Brisk Walking Increases Intestinal Bacteroides in Healthy Elderly Women. *Nutrients* **2019**, *11*, doi:10.3390/nu11040868.
43. Allen, J.M.; MAILING, L.J.; NIEMIRO, G.M.; MOORE, R.; COOK, M.D.; WHITE, B.A.; HOLSCHER, H.D.; WOODS, J.A. Exercise Alters Gut Microbiota Composition and Function in Lean and Obese Humans. *Medicine & Science in Sports & Exercise* **2018**, *50*, doi:10.1249/MSS.0000000000001495.
44. Munukka, E.; Ahtiainen, J.P.; Puigbó, P.; Jalkanen, S.; Pahkala, K.; Keskitalo, A.; Kujala, U.M.; Pietilä, S.; Hollmén, M.; Elo, L., et al. Six-Week Endurance Exercise Alters Gut Metagenome That Is not Reflected in Systemic Metabolism in Over-weight Women. *Frontiers in Microbiology* **2018**, *9*, doi:10.3389/fmicb.2018.02323.
45. Zhao, X.; Zhang, Z.; Hu, B.; Huang, W.; Yuan, C.; Zou, L. Response of Gut Microbiota to Metabolite Changes Induced by Endurance Exercise. *Frontiers in Microbiology* **2018**, *9*, doi:10.3389/fmicb.2018.00765.
46. Taniguchi, H.; Tanisawa, K.; Sun, X.; Kubo, T.; Hoshino, Y.; Hosokawa, M.; Takeyama, H.; Higuchi, M. Effects of short-term endurance exercise on gut microbiota in elderly men. *Physiological Reports* **2018**, *6*, doi:10.14814/phy2.13935.
47. Resende, A.S.; Leite, G.S.F.; Junior, A.H.L. Changes in the Gut Bacteria Composition of Healthy Men with the Same Nutritional Profile Undergoing 10-Week Aerobic Exercise Training: A Randomized Controlled Trial. *Nutrients* **202**, *13*, doi:10.3390/nu13082839.
48. Shukla, S.K.; Cook, D.; Meyer, J.; Vernon, S.D.; Le, T.; Clevidence, D.; Robertson, C.E.; Schrodi, S.J.; Yale, S.; Frank, D.N. Changes in Gut and Plasma Microbiome following Exercise Challenge in Myalgic Encephalomyelitis/Chronic Fatigue Syndrome (ME/CFS). *PLoS ONE* **2015**, *10*, doi:10.1371/journal.pone.0145453.
49. Motiani, K.K.; COLLADO, M.C.; ESKELINEN, J.-J.; VIRTANEN, K.A.; LÖYTTYNIEMI, E.; SALMINEN, S.; NUUTILA, P.; KALLIOKOSKI, K.K.; HANNUKAINEN, J.C. Exercise Training Modulates Gut Microbiota Profile and Improves Endotoxemia. *Medicine and Science in Sports and Exercise* **2020**, *52*, doi:10.1249/MSS.0000000000002112.
50. Mahdieh, M.S.; Maryam, J.; Bita, B.; Neda, F.; Motahare, M.; Mahboobeh, B.; S, Q.L.; Behrooz, S.K. A pilot study on the relationship between Lactobacillus, Bifidibactrium counts and inflammatory factors following exercise training. *Archives of Physiology and Biochemistry* **2023**, *129*, doi:10.1080/13813455.2021.1871763.

51. Verheggen, R.J.H.M.; Konstanti, P.; Smidt, H.; Hermus, A.R.M.M.; Thijssen, D.H.J.; Hopman, M.T.E. Eight-week exercise training in humans with obesity: Marked improvements in insulin sensitivity and modest changes in gut microbiome. *Obesity (Silver Spring, Md.)* **2021**, *29*, doi:10.1002/oby.23252.
52. Zeppa, S.D.; Amatori, S.; Sisti, D.; Gervasi, M.; Agostini, D.; Piccoli, G.; Pazienza, V.; Gobbi, P.; Rocchi, M.B.L.; Sestili, P., et al. Nine weeks of high-intensity indoor cycling training induced changes in the microbiota composition in non-athlete healthy male college students. *Journal of the International Society of Sports Nutrition* **2021**, *18*, doi:10.1186/s12970-021-00471-z.
53. Warbeck, C.; Dowd, A.J.; Kronlund, L.; Parmar, C.; Daun, J.T.; Wytsma-Fisher, K.; Millet, G.Y.; Schick, A.; Reimer, R.A.; Fung, T., et al. Feasibility and effects on the gut microbiota of a 12-week high-intensity interval training plus lifestyle education intervention on inactive adults with celiac disease. *Applied Physiology, Nutrition, and Metabolism* **2020**, *46*, doi:10.1139/apnm-2020-0459.
54. Cheng, R.; Wang, L.; Le, S.; Yang, Y.; Zhao, C.; Zhang, X.; Yang, X.; Xu, T.; Xu, L.; Wiklund, P., et al. A randomized controlled trial for response of microbiome network to exercise and diet intervention in patients with nonalcoholic fatty liver disease. *Nature Communications* **2022**, *13*, doi:10.1038/s41467-022-29968-0.
55. Sun, S.; Lei, O.K.; Nie, J.; Shi, Q.; Xu, Y.; Kong, Z. Effects of Low-Carbohydrate Diet and Exercise Training on Gut Microbiota. *Frontiers in Nutrition* **2022**, *9*, doi:10.3389/fnut.2022.884550.
56. Sato, M.; Suzuki, Y. Alterations in intestinal microbiota in ultramarathon runners. *Scientific Reports* **2022**, *12*, doi:10.1038/s41598-022-10791-y.
57. Qiu, L.; Gong, F.; Wu, J.; You, D.; Zhao, Y.; Xu, L.; Cao, X.; Bao, F. Exercise Interventions Improved Sleep Quality through Regulating Intestinal Microbiota Composition. *International Journal of Environmental Research and Public Health* **2022**, *19*, doi:10.3390/ijerph191912385.
58. Wang, R.; Cai, Y.; Lu, W.; Zhang, R.; Shao, R.; Yau, S.-Y.; Stubbs, B.; McIntyre, R.S.; Su, K.-P.; Xu, G., et al. Exercise effect on the gut microbiota in young adolescents with subthreshold depression: A randomized psychoeducation-controlled Trial. *Psychiatry research* **2023**, *319*, 115005-115005, doi:10.1016/j.psychres.2022.115005.
59. Lkhagva, E.; Chung, H.-J.; Ahn, J.-S.; Hong, S.-T. Host Factors Affect the Gut Microbiome More Significantly than Diet Shift. *Microorganisms (Basel)* **2021**, *9*, 2520, doi:10.3390/microorganisms9122520.
60. Fukuchi, M.; Sugita, M.; Banjo, M.; Yonekura, K.; Sasuga, Y. The impact of a competitive event and the efficacy of a lactic acid bacteria-fermented soymilk extract on the gut microbiota and urinary metabolites of endurance athletes: An open-label pilot study. *PLoS ONE* **2022**, *17*, doi:10.1371/journal.pone.0262906.
61. Keohane, D.M.; Woods, T.; O'Connor, P.; Underwood, S.; Cronin, O.; Whiston, R.; O'Sullivan, O.; Cotter, P.; Shanahan, F.; Molloy, M.G.M. Four men in a boat: Ultra-endurance exercise alters the gut microbiome. *Journal of science and medicine in sport* **2019**, *22*, 1059-1064, doi:10.1016/j.jsams.2019.04.004.
62. Bielik, V.; Hric, I.; Ugrayová, S.; Kubáňová, L.; Putala, M.; Grznár, Ľ.; Penesová, A.; Havranová, A.; Šardzíková, S.; Grendar, M., et al. Effect of High-intensity Training and Probiotics on Gut Microbiota Diversity in Competitive Swimmers: Randomized Controlled Trial. *Sports Medicine - Open* **2022**, *8*, doi:10.1186/s40798-022-00453-8.
63. Karl, J.P.; Margolis, L.M.; Madslien, E.H.; Murphy, N.E.; Castellani, J.W.; Gundersen, Y.; Hoke, A.V.; Levangie, M.W.; Kumar, R.; Chakraborty, N., et al. Changes in intestinal microbiota composition and metabolism coincide with increased intestinal permeability in young adults under prolonged physiological stress. *American journal of physiology: Gastrointestinal and liver physiology* **2017**, *312*, G559-G571, doi:10.1152/ajpgi.00066.2017.
64. Tabone, M.; Bressa, C.; García-Merino, J.A.; Moreno-Pérez, D.; Van, E.C.; Castelli, F.A.; Fenaille, F.; Larrosa, M. The effect of acute moderate-intensity exercise on the serum and fecal metabolomes and the gut microbiota of cross-country endurance athletes. *Scientific Reports* **2021**, *11*, doi:10.1038/s41598-021-82947-1.
65. Liu, Y.; Wang, Y.; Ni, Y.; Cheung, C.K.Y.; Lam, K.S.L.; Wang, Y.; Xia, Z.; Ye, D.; Guo, J.; Tse, M.A., et al. Gut Microbiome Fermentation Determines the Efficacy of Exercise for Diabetes Prevention. *Cell metabolism* **2020**, *31*, 77-91.e75, doi:10.1016/j.cmet.2019.11.001.
66. Quiroga, R.; Nistal, E.; Estébanez, B.; Porras, D.; Juárez-Fernández, M.; Martínez-Flórez, S.; García-Mediavilla, M.V.; de Paz, J.A.; González-Gallego, J.; Sánchez-Campos, S., et al. Exercise training modulates the gut microbiota profile and impairs inflammatory signaling pathways in obese children. *Experimental & molecular medicine* **2020**, *52*, 1048-1061, doi:10.1038/s12276-020-0459-0.

67. Zhong, F.; Wen, X.; Yang, M.; Lai, H.-Y.; Momma, H.; Cheng, L.; Sun, X.; Nagatomi, R.; Huang, C. Effect of an 8-week Exercise Training on Gut Microbiota in Physically Inactive Older Women. *International journal of sports medicine* **2021**, *42*, 610-623, doi:10.1055/a-1301-7011.
68. Erlandson, K.M.; Liu, J.; Johnson, R.; Dillon, S.; Jankowski, C.M.; Kroehl, M.; Robertson, C.E.; Frank, D.N.; Tuncil, Y.; Higgins, J., et al. An exercise intervention alters stool microbiota and metabolites among older, sedentary adults. *Therapeutic advances in infectious disease* **2021**, *8*, 204993612110270, doi:10.1177/20499361211027067.
69. Cronin, O.; Barton, W.; Skuse, P.; Penney, N.C.; Garcia-Perez, I.; Murphy, E.F.; Woods, T.; Nugent, H.; Fanning, A.; Melgar, S., et al. A Prospective Metagenomic and Metabolomic Analysis of the Impact of Exercise and/or Whey Protein Supplementation on the Gut Microbiome of Sedentary Adults. *mSystems* **2018**, *3*, doi:10.1128/mSystems.00044-18.
70. Huber, Y.; Pfirrmann, D.; Gebhardt, I.; Labenz, C.; Gehrke, N.; Straub, B.K.; Ruckes, C.; Bantel, H.; Belda, E.; Clément, K., et al. Improvement of non-invasive markers of NAFLD from an individualised, web-based exercise program. *Alimentary pharmacology & therapeutics* **2019**, *50*, 930-939, doi:10.1111/apt.15427.
71. Mokhtarzade, M.; Molanouri Shamsi, M.; Abolhasani, M.; Bakhshi, B.; Sahraian, M.A.; Quinn, L.S.; Negaresh, R. Home-based exercise training influences gut bacterial levels in multiple sclerosis. *Complementary therapies in clinical practice* **2021**, *45*, 101463-101463, doi:10.1016/j.ctcp.2021.101463.
72. Torquati, L.; Gajanand, T.; Cox, E.R.; Willis, C.R.G.; Zaugg, J.; Keating, S.E.; Coombes, J.S. Effects of exercise intensity on gut microbiome composition and function in people with type 2 diabetes. *European journal of sport science* **2023**, *23*, 530-541, doi:10.1080/17461391.2022.2035436.
73. Wei, S.; Brejnrod, A.D.; Trivedi, U.; Mortensen, M.S.; Johansen, M.Y.; Karstoft, K.; Vaag, A.A.; Ried-Larsen, M.; Sørensen, S.J. Impact of intensive lifestyle intervention on gut microbiota composition in type 2 diabetes: a post-hoc analysis of a randomized clinical trial. *Gut Microbes* **2022**, *14*, doi:10.1080/19490976.2021.2005407.
74. Barton, W.; Cronin, O.; Garcia-Perez, I.; Whiston, R.; Holmes, E.; Woods, T.; Molloy, C.B.; Molloy, M.G.; Shanahan, F.; Cotter, P.D., et al. The effects of sustained fitness improvement on the gut microbiome: A longitudinal, repeated measures case-study approach. *Translational sports medicine* **2021**, *4*, 174-192, doi:10.1002/tsm2.215.
75. Fart, F.; Rajan, S.K.; Wall, R.; Rangel, I.; Ganda-Mall, J.P.; Tingö, L.; Brummer, R.J.; Repsilber, D.; Schoultz, I.; Lindqvist, C.M. Differences in Gut Microbiome Composition between Senior Orienteering Athletes and Community-Dwelling Older Adults. *Nutrients* **2020**, *12*, doi:10.3390/nu12092610.
76. Liang, R.; Zhang, S.; Peng, X.; Yang, W.; Xu, Y.; Wu, P.; Chen, J.; Cai, Y.; Zhou, J. Characteristics of the gut microbiota in professional martial arts athletes: A comparison between different competition levels. *PLoS ONE* **2019**, *14*, doi:10.1371/journal.pone.0226240.
77. Petersen, L.M.; Bautista, E.J.; Nguyen, H.; Hanson, B.M.; Chen, L.; Lek, S.H.; Sodergren, E.; Weinstock, G.M. Community characteristics of the gut microbiomes of competitive cyclists. *Microbiome* **2017**, *5*, doi:10.1186/s40168-017-0320-4.
78. Morishima, S.; Aoi, W.; Kawamura, A.; Kawase, T.; Takagi, T.; Naito, Y.; Tsukahara, T.; Inoue, R. Intensive, prolonged exercise seemingly causes gut dysbiosis in female endurance runners. *Journal of Clinical Biochemistry and Nutrition* **2021**, *68*, 253-258, doi:10.3164/jcbn.20-131.
79. Mörkl, S.; Lackner, S.; Müller, W.; Gorkiewicz, G.; Kashofer, K.; Oberascher, A.; Painold, A.; Holl, A.; Holzer, P.; Meinitzer, A., et al. Gut microbiota and body composition in anorexia nervosa inpatients in comparison to athletes, overweight, obese, and normal weight controls. *International Journal of Eating Disorders* **2017**, *50*, doi:10.1002/eat.22801.
80. O'Donovan, C.M.; Madigan, S.M.; Garcia-Perez, I.; Rankin, A.; O' Sullivan, O.; Cotter, P.D. Distinct microbiome composition and metabolome exists across subgroups of elite Irish athletes. *Journal of science and medicine in sport* **2020**, *23*, 63-68, doi:10.1016/j.jsams.2019.08.290.
81. Šoltys, K.; Lendvorský, L.; Hric, I.; Baranovičová, E.; Penesová, A.; Mikula, I.; Bohmer, M.; Budiš, J.; Vávrová, S.; Grones, J., et al. Strenuous Physical Training, Physical Fitness, Body Composition and Bacteroides to Prevotella Ratio in the Gut of Elderly Athletes. *Frontiers in Physiology* **2021**, *12*, doi:10.3389/fphys.2021.670989.
82. Han, M.; Yang, K.; Yang, P.; Zhong, C.; Chen, C.; Wang, S.; Lu, Q.; Ning, K. Stratification of athletes' gut microbiota: the multifaceted hubs associated with dietary factors, physical characteristics and performance. *Gut Microbes* **2020**, *12*, doi:10.1080/19490976.2020.1842991.

83. Jang, L.-G.; Choi, G.; Kim, S.-W.; Kim, B.-Y.; Lee, S.; Park, H. The combination of sport and sport-specific diet is associated with characteristics of gut microbiota: an observational study. *Journal of the International Society of Sports Nutrition* **2019**, *16*, doi:10.1186/s12970-019-0290-y.
84. Kulecka, M.; Fraczek, B.; Mikula, M.; Zeber-Lubecka, N.; Karczmarski, J.; Paziewska, A.; Ambrozkiewicz, F.; Jagusztyn-Krynicka, K.; Cieszczyk, P.; Ostrowski, J. The composition and richness of the gut microbiota differentiate the top Polish endurance athletes from sedentary controls. *Gut Microbes* **2020**, *11*, doi:10.1080/19490976.2020.1758009.
85. Xu, Y.; Zhong, F.; Zheng, X.; Lai, H.-Y.; Wu, C.; Huang, C. Disparity of Gut Microbiota Composition Among Elite Athletes and Young Adults With Different Physical Activity Independent of Dietary Status: A Matching Study. *Frontiers in Nutrition* **2022**, *9*, doi:10.3389/fnut.2022.843076.
86. Grosicki, G.J.; Durk, R.P.; Bagley, J.R. Rapid gut microbiome changes in a world-class ultramarathon runner. *Physiological reports* **2019**, *7*, e14313-n/a, doi:10.14814/phy2.14313.
87. Hintikka, J.E.; Munukka, E.; Valtonen, M.; Luoto, R.; Ihalainen, J.K.; Kallonen, T.; Waris, M.; Heinonen, O.J.; Ruuskanen, O.; Pekkala, S. Gut Microbiota and Serum Metabolome in Elite Cross-Country Skiers: A Controlled Study. *Metabolites* **2022**, *12*, 335, doi:10.3390/metabo12040335.
88. Harnett, J.E.; Pyne, D.B.; McKune, A.J.; Penm, J.; Pumpa, K.L. Probiotic supplementation elicits favourable changes in muscle soreness and sleep quality in rugby players. *Journal of science and medicine in sport* **2021**, *24*, 195-199, doi:10.1016/j.jsams.2020.08.005.
89. Mazur-Kurach, P.; Frączek, B.; Klimek, A.T. Does Multi-Strain Probiotic Supplementation Impact the Effort Capacity of Competitive Road Cyclists? *International journal of environmental research and public health* **2022**, *19*, 12205, doi:10.3390/ijerph191912205.
90. Schreiber, C.; Tamir, S.; Golan, R.; Weinstein, A.; Weinstein, Y. The effect of probiotic supplementation on performance, inflammatory markers and gastro-intestinal symptoms in elite road cyclists. *Journal of the International Society of Sports Nutrition* **2021**, *18*, 36-36, doi:10.1186/s12970-021-00432-6.
91. Strasser, B.; Geiger, D.; Schauer, M.; Gostner, J.M.; Gatterer, H.; Burtscher, M.; Fuchs, D. Probiotic Supplements Beneficially Affect Tryptophan-Kynurenine Metabolism and Reduce the Incidence of Upper Respiratory Tract Infections in Trained Athletes: A Randomized, Double-Blinded, Placebo-Controlled Trial. *Nutrients* **2016**, *8*, 752-752, doi:10.3390/nu8110752.
92. Toohey, J.C.; Townsend, J.R.; Johnson, S.B.; Toy, A.M.; Vantrease, W.C.; Bender, D.; Crimi, C.C.; Stowers, K.L.; Ruiz, M.D.; VanDusseldorp, T.A., et al. Effects of Probiotic (Bacillus subtilis) Supplementation During Offseason Resistance Training in Female Division I Athletes. *Journal of strength and conditioning research* **2020**, *34*, 3173, doi:10.1519/JSC.0000000000002675.
93. Michalickova, D.; Minic, R.; Dikic, N.; Andjelkovic, M.; Kostic-Vucicevic, M.; Stojmenovic, T.; Nikolic, I.; Djordjevic, B. Lactobacillus helveticus Lafti L10 supplementation reduces respiratory infection duration in a cohort of elite athletes: a randomized, double-blind, placebo-controlled trial. *Applied physiology, nutrition, and metabolism* **2016**, *41*, 782-789, doi:10.1139/apnm-2015-0541.
94. Komano, Y.; Shimada, K.; Naito, H.; Fukao, K.; Ishihara, Y.; Fujii, T.; Kokubo, T.; Daida, H. Efficacy of heat-killed Lactococcus lactis JCM 5805 on immunity and fatigue during consecutive high intensity exercise in male athletes: a randomized, placebo-controlled, double-blinded trial. *Journal of the International Society of Sports Nutrition* **2018**, *15*, 39-39, doi:10.1186/s12970-018-0244-9.
95. Lamprecht, M.; Bogner, S.; Schippinger, G.; Steinbauer, K.; Fankhauser, F.; Hallstroem, S.; Schuetz, B.; Greilberger, J.F. Probiotic supplementation affects markers of intestinal barrier, oxidation, and inflammation in trained men; a randomized, double-blinded, placebo-controlled trial. *Journal of the International Society of Sports Nutrition* **2012**, *9*, 45-45, doi:10.1186/1550-2783-9-45.
96. Martarelli, D.; Verdenelli, M.C.; Scuri, S.; Cocchioni, M.; Silvi, S.; Cecchini, C.; Pompei, P. Effect of a Probiotic Intake on Oxidant and Antioxidant Parameters in Plasma of Athletes During Intense Exercise Training. *Current microbiology* **2011**, *62*, 1689-1696, doi:10.1007/s00284-011-9915-3.
97. Jäger, R.; Shields, K.A.; Lowery, R.P.; De Souza, E.O.; Partl, J.M.; Hollmer, C.; Purpura, M.; Wilson, J.M. Probiotic Bacillus coagulans GBI-30, 6086 reduces exercise-induced muscle damage and increases recovery. *PeerJ (San Francisco, CA)* **2016**, *4*, e2276-e2276, doi:10.7717/peerj.2276.
98. Roberts, J.D.; Suckling, C.A.; Peedle, G.Y.; Murphy, J.A.; Dawkins, T.G.; Roberts, M.G. An Exploratory Investigation of Endotoxin Levels in Novice Long Distance Triathletes, and the Effects of a Multi-Strain Probiotic/Prebiotic, Antioxidant Intervention. *Nutrients* **2016**, *8*, 733-733, doi:10.3390/nu8110733.
99. Salarkia, N.; Ghadamli, L.; Zaeri, F.; Sabaghian Rad, L. Effects of probiotic yogurt on performance, respiratory and digestive systems of young adult female endurance swimmers: a randomized controlled trial. *Medical journal of the Islamic Republic of Iran* **2013**, *27*, 141-146.

100. Salleh, R.M.; Kuan, G.; Aziz, M.N.A.; Rahim, M.R.A.; Rahayu, T.; Sulaiman, S.; Kusuma, D.W.Y.; Adikari, A.M.G.C.P.; Razam, M.S.M.; Radhakrishnan, A.K., et al. Effects of probiotics on anxiety, stress, mood and fitness of badminton players. *Nutrients* **2021**, *13*, 1783, doi:10.3390/nu13061783.
101. Shing, C.M.; Peake, J.M.; Lim, C.L.; Briskey, D.; Walsh, N.P.; Fortes, M.B.; Ahuja, K.D.K.; Vitetta, L. Effects of probiotics supplementation on gastrointestinal permeability, inflammation and exercise performance in the heat. *European journal of applied physiology* **2014**, *114*, 93-103, doi:10.1007/s00421-013-2748-y.
102. Pugh, J.N.; Sparks, A.S.; Doran, D.A.; Fleming, S.C.; Langan-Evans, C.; Kirk, B.; Fearn, R.; Morton, J.P.; Close, G.L. Four weeks of probiotic supplementation reduces GI symptoms during a marathon race. *European journal of applied physiology* **2019**, *119*, 1491-1501, doi:10.1007/s00421-019-04136-3.
103. West, N.P.; Pyne, D.B.; Cripps, A.W.; Hopkins, W.G.; Eskesen, D.C.; Jairath, A.; Christophersen, C.T.; Conlon, M.A.; Fricker, P.A. Lactobacillus fermentum (PCC) supplementation and gastrointestinal and respiratory-tract illness symptoms: A randomised control trial in athletes. *Nutrition journal* **2011**, *10*, 30-30, doi:10.1186/1475-2891-10-30.
104. Huang, W.C.; Wei, C.C.; Huang, C.C.; Chen, W.L.; Huang, H.Y. The beneficial effects of Lactobacillus plantarum PS128 on high-intensity, exercise-induced oxidative stress, inflammation, and performance in triathletes. *Nutrients* **2019**, *11*, 353, doi:10.3390/nu11020353.
